# Profiling of the Major Phenolic Compounds and Their Biosynthesis Genes in* Sophora flavescens* Aiton

**DOI:** 10.1155/2018/6218430

**Published:** 2018-03-01

**Authors:** Jeongyeo Lee, Jaeeun Jung, Seung-Hyun Son, Hyun-Bi Kim, Young-Hee Noh, Sung Ran Min, Kun-Hyang Park, Dae-Soo Kim, Sang Un Park, Haeng-Soon Lee, Cha Young Kim, Hyun-Soon Kim, Hyeong-Kyu Lee, HyeRan Kim

**Affiliations:** ^1^Korea Research Institute of Bioscience and Biotechnology, 125 Gwahak-ro, Yuseong-gu, Daejeon 34141, Republic of Korea; ^2^Department of Crop Science, Chungnam National University, 99 Daehak-ro, Yuseong-gu, Daejeon 34134, Republic of Korea; ^3^Korea Research Institute of Bioscience and Biotechnology, 181 Ipsin-gil, Jeongeup-si, Jeollabuk-do 56212, Republic of Korea; ^4^Korea Research Institute of Bioscience and Biotechnology, Yeongudanji-ro 30, Ochang-eup, Cheongwon-gu, Cheongju-si 28116, Republic of Korea; ^5^Systems and Bioengineering, University of Science and Technology, 217 Gajung-ro, Yuseong-gu, Daejeon 34113, Republic of Korea

## Abstract

Sophorae Radix (*Sophora flavescens *Aiton) has long been used in traditional medicine in East Asia due to the various biological activities of its secondary metabolites. Endogenous contents of phenolic compounds (phenolic acid, flavonol, and isoflavone) and the main bioactive compounds of Sophorae Radix were analyzed based on the qualitative HPLC analysis and evaluated in different organs and at different developmental stages. In total, 11 compounds were detected, and the composition of the roots and aerial parts (leaves, stems, and flowers) was significantly different. trans-Cinnamic acid and* p*-coumaric acid were observed only in the aerial parts. Large amounts of rutin and maackiain were detected in the roots. Four phenolic acid compounds (benzoic acid, caffeic acid, ferulic acid, and chlorogenic acid) and four flavonol compounds (kaempferol, catechin hydrate, epicatechin, and rutin) were higher in aerial parts than in roots. To identify putative genes involved in phenolic compounds biosynthesis, a total of 41 transcripts were investigated. Expression patterns of these selected genes, as well as the multiple isoforms for the genes, varied by organ and developmental stage, implying that they are involved in the biosynthesis of various phenolic compounds both spatially and temporally.

## 1. Introduction

Sophorae Radix (*Sophora flavescens* Aiton) belongs to the Fabaceae family and is widely distributed in Asia (especially in Korea, China, Japan, and India) and some European countries [1]. The roots of Sophorae Radix, known as “Kosam” in Korean (“Kushen” in Chinese), have been used as a functional food ingredient and traditional herbal medicine as an antipyretic, diuretic, and anthelmintic, as well as for the treatments of diarrhea, gastrointestinal hemorrhage, and eczema [2].

More than 200 compounds have been isolated and identified from Sophorae Radix, including alkaloids, flavonoids, terpenoids, and other compounds [1, 3, 4]. Among these compounds, the main active compounds are alkaloids and flavonoids [5]. Alkaloid composition and content vary between the organs of Sophorae Radix, such as the roots, stems, leaves, flowers, and seeds [6]. The 27 alkaloids have been isolated and identified from the roots, and another 20 alkaloids have been reported from the aerial parts, flowers, and seeds of Sophorae Radix [1]. The alkaloid components, such as matrine, oxymatrine, sophocarpine, and sophoridine, are rare in the plant kingdom and are largely found in* Sophora* species [7]. They have various pharmacological effects, including antimicrobial, anti-inflammatory, antiallergic, antiarrhythmia, antihepatitis, and regulation of the immune system [8–11].

Flavonoids are a class of low molecular weight phenolic compounds and are widely distributed in the plant kingdom. They have been classified into six subgroups: flavones, flavonols, flavanones, flavan-3-ols, isoflavones, and anthocyanidins [12]. The dried roots of Sophorae Radix contain flavonoids, such as kuraridin, kurarinone, isokurarinine, norkurarinone, pterocarpin, formononetin, trifolirhizin, daidzein, umbelliferone, maackiain, kuraridinol, kurarinol, neo-kurinol, and norkurarinol [13, 14]. These flavonoid compounds have diverse biological functions, such as flower coloring, UV light protection, auxin transport, defense, allelopathy, anticancer, antiasthmatic, anti-inflammatory, and antimicrobial activities [5, 15–17].

The benefits of phenolic compounds for both plants and humans have inspired efforts to enhance their effects through genetic modification. Phenolic compounds biosynthetic pathway and its regulation have been almost completely elucidated in plants [18]. Many of the structural and regulatory genes have been characterized in* Arabidopsis thaliana*, tomato, maize, tobacco, soybean, alfalfa, and petunia [19]. These genes were genetically modified in* E. coli*, yeast, and plants to enhance flavonoid content [19, 20].* CHI*, an early enzyme of the flavonols biosynthesis pathway, was found to be the key gene for increasing flavonol production [21]. Overexpression of petunia* CHI* and* CHS* genes in tomatoes was sufficient for the accumulation of rutin and naringenin contents, respectively. However, RNAi inhibition of the tomato* CHS1* gene resulted in a strong reduction of both naringenin and quercetin levels [22]. Overexpression of* F3H* and* FLS* showed no effects on flavonoid levels compared to nontransgenic controls, but* F3H* RNAi tomatoes resulted in a 20% decrease in wild-type rutin levels [21, 23]. Seed oil extracts overexpressed with multigene (*CHS*,* CHI*, and* DFR*) from petunia into flax exhibited higher levels of quercetin (46–90%), kaempferol (70–83%), and anthocyanin (198%) than the control [24]. A clear reduction in quercetin-3-rutinoside levels was obtained by introducing* FLS* RNAi construct to tomatoes [23]. In soybeans, RNAi silencing of* IFS* gene resulted in a 95% reduction in total isoflavonoids in the transgenic roots [25]. In maize, downregulated* COMT* expression by RNAi silencing reduced* p*-coumaric acid content but increased ferulic acid level [26]. Previous studies have demonstrated the important roles these genes could play in the production of flavonoid components.

A recent study isolated alkaloids, flavonoids, benzofuran, and triterpenoid from the roots of Sophorae Radix. Phytochemical studies revealed that Sophorae Radix mostly contains alkaloids and flavonoids, which possess a wide range of biological activities, including anticancer, anti-inflammatory, and antibacterial properties [9, 13, 15]. However, pharmacological research has largely focused on alkaloids, and little work has been done to examine the expression analysis of flavonoid biosynthesis genes. Thus, our objective was to analyze the phenolic compounds in Sophorae Radix and identify key genes involved in the biosynthesis of phenolic acids and flavonoids. Furthermore, we aimed to examine the levels of expression in different organs and developmental stages to investigate the correlations between gene expression and phenolic compounds.

## 2. Materials and Methods

### 2.1. Plant Material

Different organs (roots, leaves, and stems) were separately harvested from Sophorae Radix collected in Damyang (Jeollanam-do, Korea) in April 2016. The height of the sampled plants was 150–200 centimeters. Leaves were grouped according to length and width, which represented different developmental stages (Table 1). Stems were divided into three categories based on diameter (Table 2). The flower samples were collected from the wild Sophorae Radix transferred to the experimental farm of the Korea Research Institute of Bioscience and Biotechnology (Daejeon, Korea) after 90 days of growing. All samples were immediately frozen in liquid nitrogen and stored at −80°C for HPLC analysis and total RNA isolation.

### 2.2. Identification of Phenolic Compounds Biosynthesis Genes in Sophorae Radix

Phenolic compounds biosynthesis genes of* Glycine max*,* Cicer arietinum*, and* Arabidopsis thaliana* were searched from NCBI GenBank (https://www.ncbi.nlm.nih.gov/) and the KEGG database (http://www.genome.jp/kegg/kegg2.html). The collected genes were subjected to a tBlastN search against our internal transcriptome database of Sophorae Radix (unpublished data) to identify their homologs in the Sophorae Radix genome. Only resultant sequences with *e*-value of <1*e*^−100^ and identity of >50% were considered as orthologous genes. In total, 21 Sophorae Radix phenolic compounds biosynthesis genes were selected, and gene-specific primers for the 41 transcript IDs were designed with Primer3 (v. 0.4.0) (http://bioinfo.ut.ee/primer3-0.4.0/primer3/) and used for semiquantitative RT-PCR analysis. The accession numbers of the genes and primer sets used in this study are listed in Supplementary Tables 2 and 3, respectively.

### 2.3. Total RNA Extraction and Semiquantitative RT-PCR

Total RNA was isolated using the Trizol reagent (Gibco-BRL) according to the manufacturer's protocol. Total RNA (1 *μ*g) was reverse-transcribed by a ReverTra Ace-*α* Kit (TOYOBO) according to the manufacturer's protocol. The cDNA was diluted 10-fold, and 1 *μ*l of diluted cDNA was used in a 20 *μ*l PCR reaction. Semiquantitative RT-PCR was performed using gene-specific primers and actin11* (SfACT11)* as the housekeeping gene. PCR was performed with a 5 min denaturation at 94°C, followed by 28 cycles of 94°C for 30 sec each at 55°C, followed by 72°C for 1 min. PCR products were analyzed by using 1.2% agarose gel, stained with ethidium bromide (EtBr), and visualized under ultraviolet light by Gel Doc™ XR+ image system (Bio-Rad). The densitometry data for band intensities was generated by analyzing the gel images using the Image Lab™ Software (Bio-Rad).

### 2.4. Extraction and Quantitative HPLC Analysis of Phenolic Compounds in Sophorae Radix

Phenolic compounds extraction was performed according to previously described methods with some modification [27]. Freeze-dried samples (100 mg) were extracted using 3 ml of MeOH. Samples were then vortexed at 24°C for 5 min and stored at 60°C for 30 min. After centrifugation at 4,000 rpm for 5 min, the supernatant was filtered through a 0.45 *μ*m PTFE syringe filter (Advantec DISMIC-13HP, Toyo Roshi Kaisha) for HPLC analysis. The HPLC analysis of flavonoids was performed on a Futecs model NS-4000 HPLC apparatus equipped with a C18 column (250 mm × 4.6 mm, 5 *μ*m, RStech). The mobile phase was gradient prepared from mixtures of acetonitrile and 0.15% acetic acid; the column was maintained at 30°C. The flow rate was 1.0 ml/min, and the injection volume was 20 *μ*l. Different compounds were quantified on the basis of peak areas and calculated as equivalents of representative standard compounds. All samples were run in triplicate.

## 3. Results and Discussion

### 3.1. Analysis of Phenolic Compound Contents in Sophorae Radix

**Figure 1:**
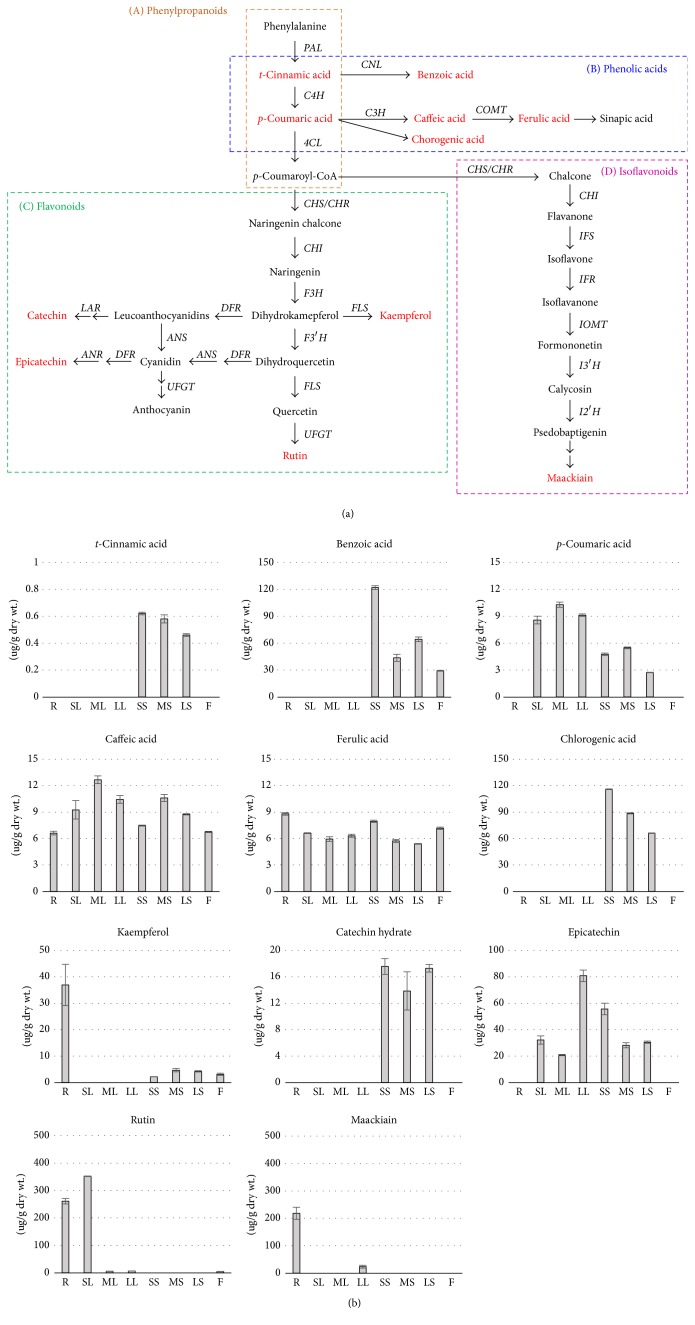
(a) A schematic presentation of general flavonoid biosynthetic pathway. Multiple arrows indicate two or more steps in the pathway, and flavonoids that analyzed in this study are highlighted in red.* PAL*, phenylalanine ammonia lyase;* CNL*, cinnamoyl-CoA ligase;* C4H*, cinnamic acid 4-hydroxylase;* C3H*,* p*-coumaroyl ester 3-hydroxylase;* COMT*, caffeic acid 3-*O*-methyltransferase;* 4CL*, 4-coumaroyl:CoA-ligase;* CHS*, chalcone synthase;* CHR*, chalcone reductase;* CHI*, chalcone isomerase;* F3H*, flavanone 3-hydroxylase;* F3*′*H*, flavanone 3′-hydroxylase;* FLS*, flavonol synthase;* UFGT*, UDP glucose: flavonoid-3-*O*-glucosyltransferase;* FLS*, flavonol synthase;* DFR*, dihydroflavonol 4-reductase;* LAR*, leucoanthocyanidin reductase;* ANS*, anthocyanidin synthase;* ANR*, anthocyanidin reductase;* IFS*, isoflavone synthase;* IOMT*, isoflavone O-methyltransferase;* I3*′*H*, isoflavone 3′-hydroxylase;* I2*′*H*, isoflavone 2′-hydroxylase. (b) Contents of flavonoid compound in roots, leaves, stems, and flowers of Sophorae Radix (ug/g dry weight). R, roots; SL, small leaves; ML, medium leaves; LL, large leaves; SS, small stems; MS, medium stems; LS, large stems; F, flowers.

Previous studies have reported abundant phenolic compounds in Sophorae Radix, such as rutin, quercetin, kaempferol, kurarinol, and maackiain [13, 28–30]. To investigate the amounts of phenolic compounds in the various organs and developmental stages, we analyzed different sized leaves and stems of Sophorae Radix with its roots (Tables 1 and 2). In total, 11 compounds were detected in Sophorae Radix: 6 phenolic acids (*t*-cinnamic acid, benzoic acid,* p*-coumaric acid, caffeic acid, ferulic acid, and chlorogenic acid), 4 flavonols (kaempferol, catechin hydrate, epicatechin, and rutin), and 1 isoflavone (maackiain) (Figure 1(b) and Supplementary Table 1). Caffeic acid and ferulic acid were detected in all four organs and ranged within 6.62 ± 0.21 *μ*g/g dry wt. (R)~12.67 ± 0.44 *μ*g/g dry wt. (ML) and 5.40 ± 0.04 *μ*g/g dry wt. (LS)~8.79 ± 0.14 *μ*g/g dry wt. (R), respectively. All detected components were found in the stems except rutin and maackiain, which were the major components of Sophorae Radix. Only the stems contained tissue-specific compounds, including* t*-cinnamic acid, chlorogenic acid, and catechin hydrate. Among the six phenolic acids, benzoic acid and chlorogenic acid were the most abundant compounds detected in the stems and the flowers or stems, respectively. Those were lowest in older tissues (MS, LS) and highest in SS (121.83 ± 2.16 *μ*g/g dry wt. and 115.94 ± 0.46 *μ*g/g dry wt., resp.). Benzoic acid was the major phenolic acid compound in the flowers (F, 29.12 ± 0.48 *μ*g/g dry wt.). trans-Cinnamic acid was detected only in stems at minimal levels and ranged within 0.46 ± 0.01–0.62 ± 0.01 *μ*g/g dry wt. Additionally,* p*-coumaric acid occurred in higher levels in the leaves (8.57 ± 0.43–10.28 ± 0.29 *μ*g/g dry wt.) than in the stems (2.73 ± 0.03–5.47 ± 0.10 *μ*g/g dry wt.); it was not detected in the roots or flowers. Although caffeic, chlorogenic, ferulic, and* p*-coumaric acids are derived from* t*-cinnamic acid [31], we found that* t*-cinnamic acid was detected only in the stems at minimal levels (Figure 1(b) and Supplementary Table 1). Flavonoids were differentially taken up and transported long distances to distal tissues via cell-to-cell movement in* Arabidopsis thaliana* [32]. Thus,* t*-cinnamic acid as a precursor to other phenolic acids might move from the stem to other organs in Sophorae Radix. Among four flavonol compounds, rutin was detected at high levels in the roots and small leaves (261.03 ± 10.07 *μ*g/g dry wt. and 352 ± 1.02 *μ*g/g dry wt., resp.), while ML, LL, and F exhibited low levels of rutin (5.46 ± 0.15 *μ*g/g dry wt., 6.3 ± 0.08 *μ*g/g dry wt., and 4.8 ± 0.20 *μ*g/g dry wt., resp.). Kaempferol was observed in the roots, stems, and flowers with a range of 2.23 ± 0.03 *μ*g/g dry wt. (SS)~36.91 ± 7.82 *μ*g/g dry wt. (R). Epicatechin occurred in the leaves and stems, where older leaves had a greater accumulation and younger stems had a lower accumulation. Catechin hydrate was present in stems alone and showed similar amounts among the SS, MS, and LS (Figure 1(b) and Supplementary Table 1). The isoflavone maackiain was detected in the roots at high levels (218.17 ± 22.04 *μ*g/g dry wt.) and in the large leaves (23.59 ± 5.14 *μ*g/g dry wt.). 32 phenolic compounds from the stems and leaves of Sophorae Radix by metabolite profiling and demonstrated that the composition of the roots and the aerial parts were significantly different [33]. Compared to the roots, the stems and leaves of Sophorae Radix possessed more isoflavonoids than prenylated flavonoids [6]. In the present study, the amounts of six phenolic acid compounds (*t*-cinnamic acid,* p*-coumaric acid, benzoic acid, caffeic acid, ferulic acid, and chlorogenic acid) and four flavonoid compounds (kaempferol, catechin hydrate, epicatechin, and rutin) were 44.31-fold and 2.28-fold higher in aerial parts than roots, respectively. Isoflavonoid content (maackiain) was 9.24-fold higher in R than aerial parts significantly (Supplementary Table 1).

### 3.2. Candidate Genes Involved in Phenolic Compounds Biosynthesis of Sophorae Radix

The phenolic compounds biosynthetic pathway has been extensively characterized in plants (Figure 1(a)). According to the chemical structures of phenolic compounds, genes for the phenolic compounds biosynthesis pathway can be classified into four groups: phenylpropanoids, phenolic acids, flavonols, and isoflavones [34–36]. To identify putative genes involved in Sophorae Radix, we collected mRNA sequences of 21 phenolic compounds biosynthesis genes from* Glycine max* and* Cicer arietinum *(Fabaceae), as well as* Arabidopsis thaliana*. We obtained a total of 41 transcripts as the phenolic compounds biosynthesis genes in Sophorae Radix using our internal transcriptome database of it. Multiple copies of the transcripts were detected for* SfC4H*,* SfCOMT*,* SfCHR*,* SfFLS*,* SfIFR*,* SfIOMT*, and* SfI3*′*H* (Supplementary Table 2), suggesting that Sophorae Radix uses multiple isoforms of these phenolic compounds biosynthesis genes and that its diverse phenolic compounds vary in chemical, physical, and biochemical properties. Previous studies demonstrated that most flavonoid biosynthesis genes in legumes, such as* Trifolium subterraneum *and* Medicago truncatula,* were multigene families [37], while most of the genes in* Arabidopsis thaliana* exist in single copies [19].

### 3.3. Expression Analysis of Phenolic Compounds Biosynthesis Genes in Different Organs and Developmental Stages of Sophorae Radix

**Figure 2:**
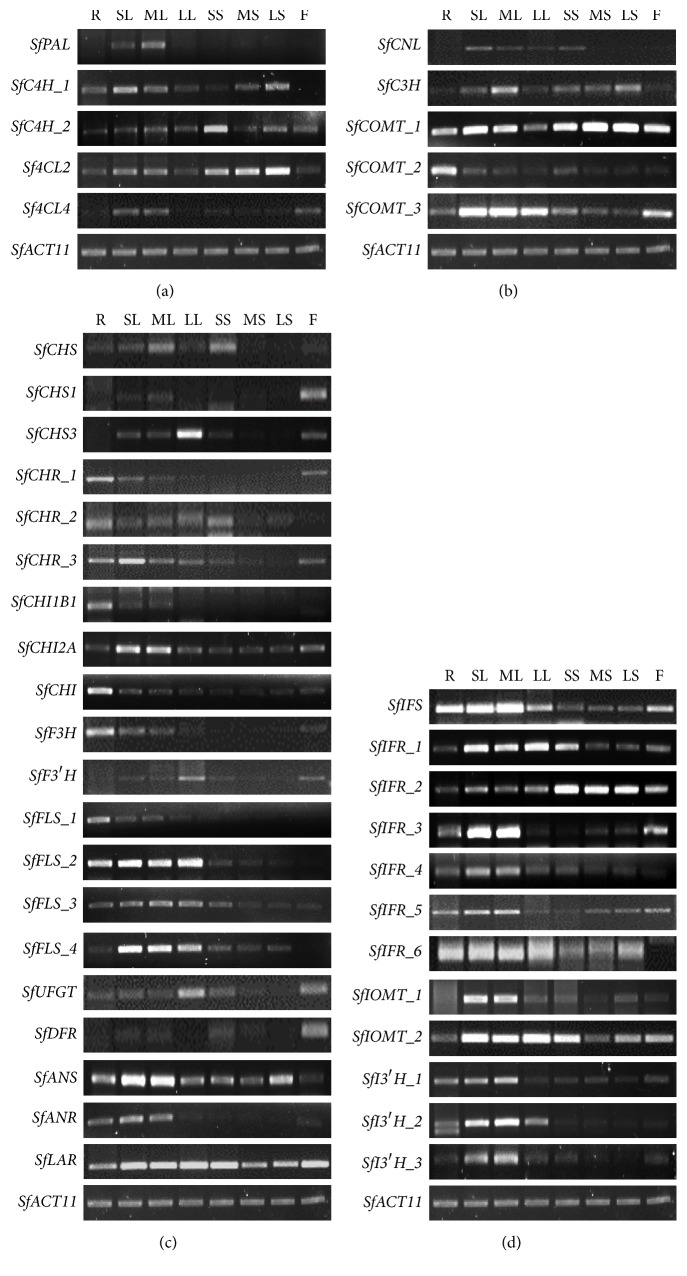
Semiquantitative RT-PCR transcript analysis of the flavonoid biosynthesis genes in root, leaves, stems, and flower of Sophorae Radix. (a) Phenylpropanoid biosynthetic genes. (b) Phenolic acid biosynthetic genes. (c) Flavonoid biosynthetic genes. (d) Isoflavonoid biosynthetic genes. Transcript levels were determined after normalization with* Actin11* as the reference gene. R, root; SL, small leaf; ML, medium leaf; LL, large leaf; SS, small stem; MS, medium stem; LS, large stem; F, flower.

To investigate the expression patterns of phenolic compounds biosynthesis genes, semiquantitative RT-PCR was performed in different organs of Sophorae Radix (roots, leaves, stems, and flowers) and during different developmental stages (Figure 2 and Supplementary Figure 1). The highest levels of* SfPAL*, the first enzyme in the phenylpropanoid biosynthetic pathway, was were expressed in ML, while other organs expressed low levels (Figure 2(a)). Transcript levels of* SfC4H* and* Sf4CL*, the genes encoding central enzymes on the phenylpropanoid pathway, varied among organs and developmental stages.* SfC4H_1* was expressed in roots, leaves, and stems; it decreased as leaves grew and increased as stems grew. The other transcript for* SfC4H*,* SfC4H_2*, was detected in all organs and expressed the highest level in SS.* Sf4CL2* exhibited higher expression levels in stems, and similar amounts were expressed in roots, leaves, and flowers.* Sf4CL4* was low in all organs, although higher expression levers were detected in SL, ML, and F (Figure 2(a)). A previous study reported that the expression of* PAL* and* C4H* in tea leaves were in accordance with the catechin concentration [38, 39]. However, transcript levels of* SfPAL* and* SfC4H* did not correlate to flavonoid contents in our results (Figures 1(b) and 2(a)). Phosphorylation of PAL in French bean [40] and ubiquitination of PAL by KFB proteins in* Arabidopsis thaliana* were discovered [33]. Conservation of the phosphorylation site in PAL from diverse species suggests that phosphorylation of PAL may be a ubiquitous regulatory mechanism in higher plants; therefore the expression of* SfPAL* and* SfC4H* might be regulated by phosphorylation and other posttranscriptional modifications.

We analyzed the expression patterns of five transcripts related to phenolic acid biosynthesis (Figure 2(b)). Very low* SfCNL* transcription levels were detected in leaves and SS, whereas* SfC3H* was expressed in leaves (highest in ML) and stems (highest in LS). Different expression levels in each organ suggested that they were specific for the biosynthesis of various phenolic acids in different organs. Among the three* SfCOMT* transcripts,* SfCOMT_1* was predominantly expressed in all organs, except in LL that showed high transcription levels of* SfCOMT_3* (Figure 2(b)).* SfCOMT_2* exhibited the highest transcription level in R compared to the other organs, while* SfCOMT_3* was detected at higher levels in the leaves and flowers. The unique expression patterns of* SfCOMT* transcripts among organs could alter the accumulation pattern of phenolic acid compounds. It was reported that the reduced* COMT* expression contributed to lignin,* p*-coumaric acid, and ferulic acid content in maize [26]. However, such correlations were not detected in our results.

The 20 transcripts were identified (Figure 2(c)) as flavonoid biosynthesis genes from our in-house transcriptome data (in progress). The expression patterns of three* SfCHS* isoforms, the first enzyme specific for the flavonoid pathway, were distinguishable by organs or developmental stages.* SfCHS* transcripts were substantially present in ML and SS compared to other samples.* SfCHS1 *and* SfCHS3* transcripts were most abundant in F and LL, respectively. Three homologs of* SfCHR* also showed distinct expression patterns compared to each other. R even exhibited all three transcriptions, and the expression of* SfCHR_2* was at a very low level compared to the other homologs in F. The leaves showed all three homolog expressions with decreased expression pattern by aging for* SfCHR_1* and* SfCHR_3* and vice versa for* SfCHR_2*. In stems,* SfCHR_1* was expressed at very low levels;* SfCHR_2* showed the major expression especially in SS and the expression of* SfCHR_3* with lower level than in leave was decreased by aging. Transcript levels of* SfCHI1B1* and* SfCHI*, which catalyze the second step of the flavonoid biosynthetic pathway, were notably low in all organs except the roots. The* SfCHI2A* transcript was highly abundant in SL and ML compared to other organs (2.6- and 2.4-fold higher than in R), respectively.* SfF3H* and* SfF3*′*H* were highly expressed in R and LL, respectively. Of the four* SfFLS* transcripts,* SfFLS_1* and* SfFLS_2* were highly expressed in the roots and leaves, while* SfFLS_3* and* SfFLS_4* were highly expressed in the leaves alone. Overall,* SfF3H*,* SfF3*′*H*, and* SfFLS* transcripts were highly expressed in the roots and leaves (Figure 2(c)). As depicted in Figure 1(b), the rutin contents exist in high amounts in R and SL, implying that the accumulation of rutin may be related to the expression of these genes. The* Arabidopsis thaliana* genome contains five* AtFLS*, and this redundancy was explained by* Arabidopsis thaliana* using multiple isoforms of* FLS* with different substrate specificities to mediate the production of flavonoids in a tissue-specific manner [41]. In the present study, the multiple isoforms of flavonoid biosynthesis genes in Sophorae Radix demonstrated organ-specific expression patterns, implying that they might have different physiological processes for biosynthesis depending on the organ.* SfUFGT* was highly expressed in LL and F. Transcript levels of* SfDFR* were high in F but low in other organs. Transcript levels of* SfANS* in SL and ML were highest among the flavonoid biosynthesis genes. In leaves,* SfANS *transcript levels increased as stem age increased and decreased as leaf age increased. Relatively high expression of* SfLAR* was detected in all organs (Figure 2(c)). A previous study reported that flavonoid biosynthetic genes were differentially regulated by the interaction of various transcription factors, such as* TT2*,* TT8*, and* TTG1*, in* Arabidopsis thaliana* [42] and by the* MYB-bHLH-WD40* transcription factors (MBW complex) in maize [43]. In the case of rice,* OsCHI1* could interact physically with* OsF3H*,* OsF3*′*H*,* OsDFR*, and* OsANS1* by forming a flavonoid multienzyme complex [44]. It has been also asserted that a protein-protein interaction between* IFS* and* C4H* could work as a tandem anchor that tethers the enzyme complex to the endoplasmic reticulum with* CHS*,* CHR,* and* CHI* in soybean [45]. The expression patterns of our selected genes in Sophorae Radix varied depending on the organ as well as the developmental stage. This implied that the genes must interact with other complexes and that they are spatially and temporally involved in the biosynthesis of various phenolic compounds.

Among the isoflavonoid biosynthesis genes of Sophorae Radix (Figure 2(d)),* SfIFS* was expressed at the highest levels in R, SL, ML, LL, and F but at lower levels in the stems, regardless of age. Of five* SfIFR* transcripts, the highest expression of* SfIFR_1* occurred in LL.* SfIFR_2* was the most abundantly expressed* SfIFR* transcript in SS, MS, LS, and F. Among the isoflavonoid biosynthesis genes,* SfIFR_3* was the most abundant in SL and ML.* SfIFR_3*,* SfIFR_4*, and* SfIFR_5* showed sufficient expression levels in SL and ML, implying that these genes might play a role in the biosynthesis of isoflavonoids in young leaves.* SfIFR_6* was the most abundant* SfIFR *transcript in R, and similar amounts of it were expressed in SL, ML, and LL.* SfIOMT_1 *was predominantly expressed in SL and ML, while* SfIOMT_2 *was expressed in high level in SL, ML, LL, and SS. Three* SfI3*′*H* transcripts were expressed, with* SfI3*′*H*_2 as the predominant transcript in SL and ML. All three* SfI3*′*H* transcripts were rarely expressed in the stems or flowers (Figure 2(d)). These results suggested that genes in isoflavonoid biosynthesis pathway were mainly expressed in the roots and younger leaves.

## 4. Conclusions

In this study, 6 phenolic acid compounds, 4 flavonol compounds, and 1 isoflavone were evaluated in different organs and at different developmental stages of Sophorae Radix (*Sophora flavescens *Aiton). The composition of these compounds between the roots and aerial parts was significantly different. The total amounts of 6 phenolic acids (*t*-cinnamic acid, benzoic acid,* p*-coumaric acid, caffeic acid, ferulic acid, and chlorogenic acid) were 1.58-fold lower in the roots compared to the aerial parts. The total amounts of 4 flavonoids (kaempferol, catechin hydrate, epicatechin, and rutin) and 1 isoflavone (maackiain) were 1.34-fold higher in the roots than in the aerial parts. In particular, large amounts of rutin and maackiain were detected in the roots. The phenolic compounds biosynthesis genes of Sophorae Radix were identified and their expression patterns were examined in the roots, leaves, stems, and flowers. The multiple isoforms for the phenolic compounds biosynthesis genes were detected and those transcripts showed spatially and temporally specific expression patterns. These patterns could contribute to the diverse components of phenolic compounds in Sophorae Radix. Our results could be a stepping-stone for understanding the varying compositions of phenolic compounds in different organs and developmental stages in Sophorae Radix. This knowledge could also aid in the identification of the major genes for large-scale production of valuable phenolic compounds in the functional food industry.

## Figures and Tables

**Table 1:** Sizes of collected leaves of Sophorae Radix: SL, small leaves; ML, medium leaves; LL, large leaves.

	Length (cm)	Width (cm)
SL	2.1 ± 0.2	0.5 ± 0.1
ML	4.3 ± 0.2	1.3 ± 0.1
LL	5.4 ± 0.3	2.3 ± 0.2

**Table 2:** Sizes of collected stems of Sophorae Radix: SS, small stems; MS, medium stems; LS, large stems.

	Diameter (cm)
SS	0.2 ± 0.1
MS	0.5 ± 0.1
LS	1.1 ± 0.2

## Supplementary Materials

Supplementary Materials Supplementary Figure 1: relative level of the flavonoid biosynthesis genes in root, leaves, stems, and flower of Sophorae Radix. (a) Phenylpropanoid biosynthetic genes. (b) Phenolic acid biosynthetic genes. (c) Flavonoid biosynthetic genes. (d) Isoflavonoid biosynthetic genes. Transcript levels were determined after normalization with* Actin11* as the reference gene. Data are means of relative band intensities of three replicates and standard deviation (SD). R, root; SL, small leaf; ML, medium leaf; LL, large leaf; SS, small stem; MS, medium stem; LS, large stem; F, flower. Supplementary Table 1: flavonoids contents in different organs of Sophorae Radix (*μ*g/g dry weight): R, roots; SL, small leaves; ML, medium leaves; LL, large leaves; SS, small stems; MS, medium stems; LS, large stems; F, flowers. Supplementary Table 2: blast search results of flavonoid biosynthetic genes in Sophorae Radix. Supplementary Table 3: primers used in this study for semiquantitative RT-PCR of flavonoid biosynthetic genes.
