# Supplementary material for: Profiling of the Major Phenolic Compounds and Their Biosynthesis Genes in Sophora flavescens Aiton
Source: ScientificWorldJournal. 2018 Mar 1;2018:6218430. doi: 10.1155/2018/6218430 (PMC5852875; doi:10.1155/2018/6218430)
Supplement: Supplementary Materials — Supplementary Figure 1: relative level of the flavonoid biosynthesis genes in root, leaves, stems, and flower of Sophorae Radix. (a) Phenylpropanoid biosynthetic genes. (b) Phenolic acid biosynthetic genes. (c) Flavonoid biosynthetic genes. (d) Isoflavonoid biosynthetic genes. Transcript levels were determined after normalization with Actin11 as the reference gene. Data are means of relative band intensities of three replicates and standard deviation (SD). R, root; SL, small leaf; ML, medium leaf; LL, large leaf; SS, small stem; MS, medium stem; LS, large stem; F, flower. Supplementary Table 1: flavonoids contents in different organs of Sophorae Radix (μg/g dry weight): R, roots; SL, small leaves; ML, medium leaves; LL, large leaves; SS, small stems; MS, medium stems; LS, large stems; F, flowers. Supplementary Table 2: blast search results of flavonoid biosynthetic genes in Sophorae Radix. Supplementary Table 3: primers used in this study for semiquantitative RT-PCR of flavonoid biosynthetic genes. [file 6218430.f1.pdf]

(a)

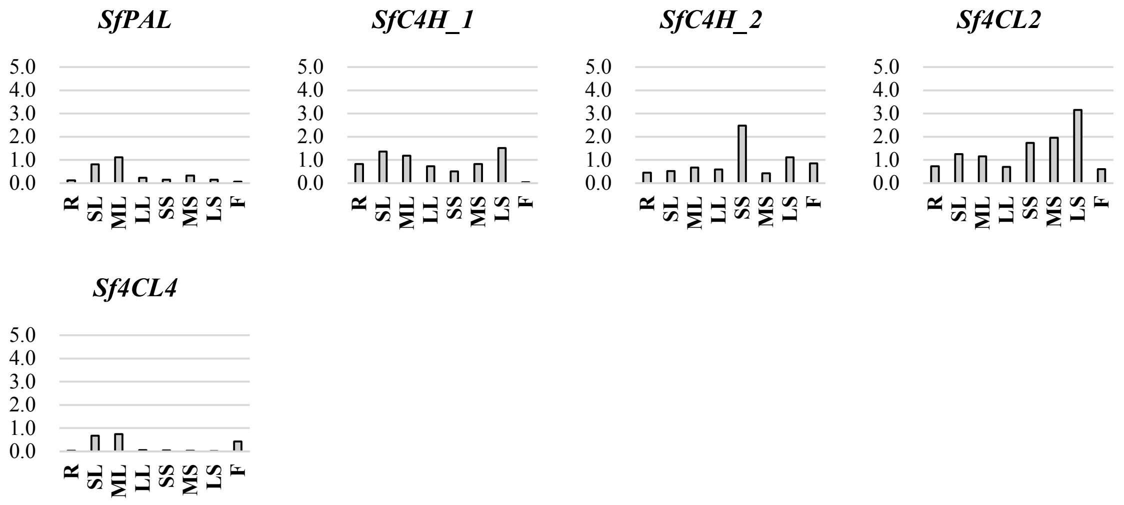
SfPAL
SfC4H_1
SfC4H_2
Sf4CL2
Sf4CL4
5.0 4.0 3.0 2.0 1.0 0.0
R SL ML LL SS MS LS F

(b)

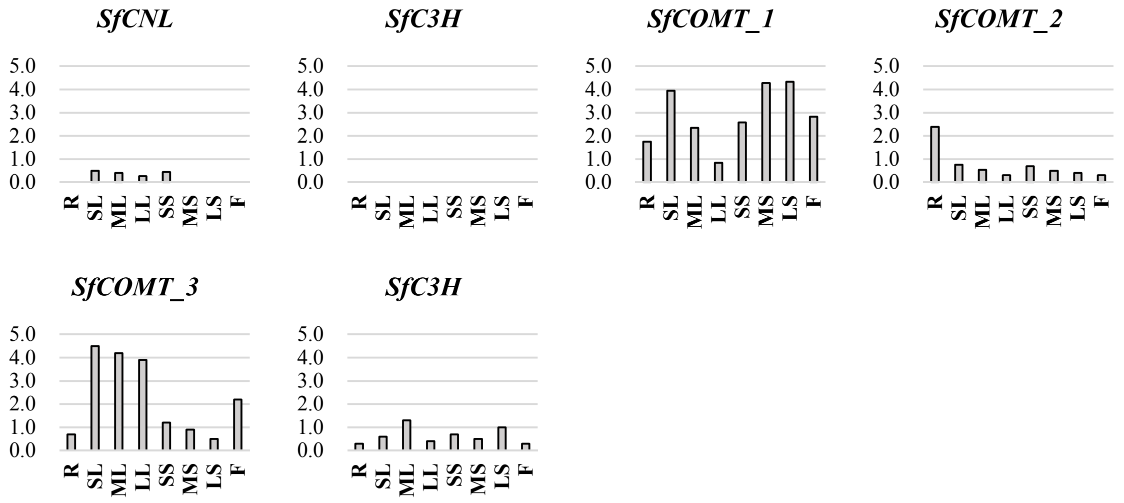
SfCNL
SfC3H
SfCOMT_1
SfCOMT_2
SfCOMT_3
SfC3H
5.0 4.0 3.0 2.0 1.0 0.0
R SL ML LL SS MS LS F

(c)

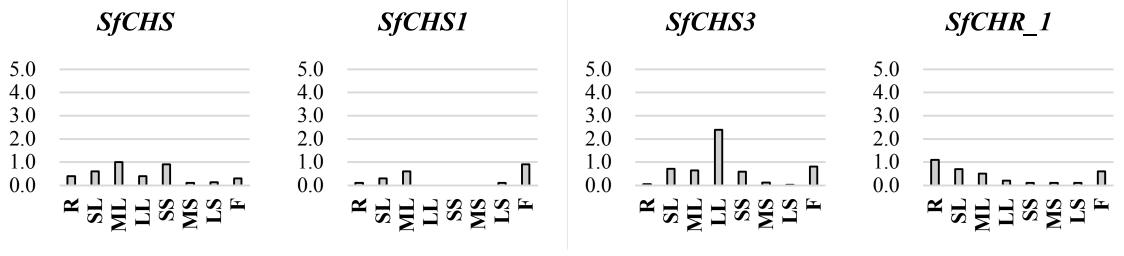
SfCHS
SfCHS1
SfCHS3
SfCHR_1
5.0 4.0 3.0 2.0 1.0 0.0
R SL ML LL SS MS LS F

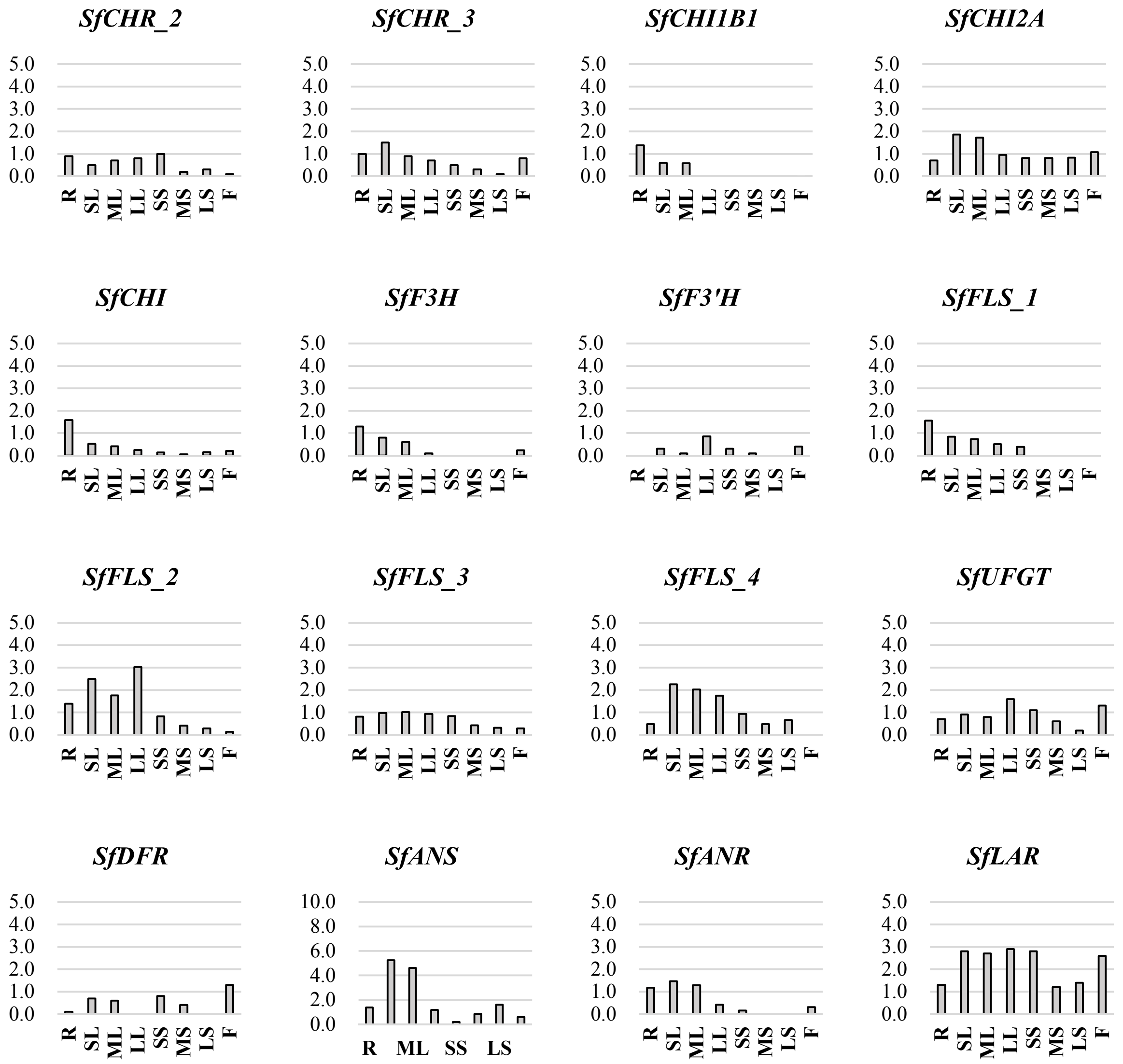
SfCHR_2
SfCHR_3
SfCHI1B1
SfCHI2A
SfCHI
SfF3H
SfF3'H
SfFLS_1
SfFLS_2
SfFLS_3
SfFLS_4
SfUFGT
SfDFR
SfANS
SfANR
SfLAR
0.0 1.0 2.0 3.0 4.0 5.0
0.0 2.0 4.0 6.0 8.0 10.0
R SL ML LL SS MS LS F

(d)

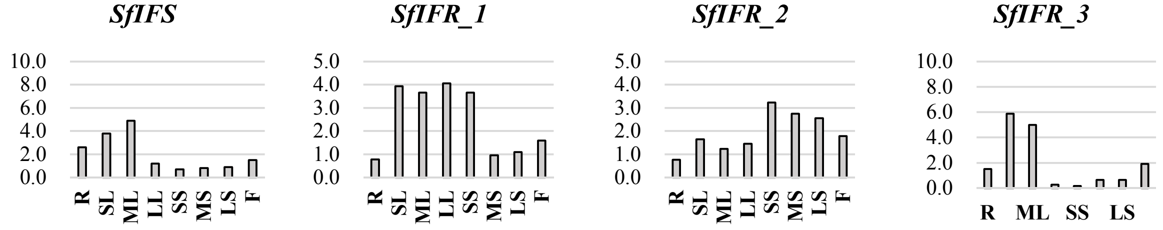
SfIFS
SfIFR_1
SfIFR_2
SfIFR_3
0.0 2.0 4.0 6.0 8.0 10.0
0.0 1.0 2.0 3.0 4.0 5.0
R SL ML LL SS MS LS F

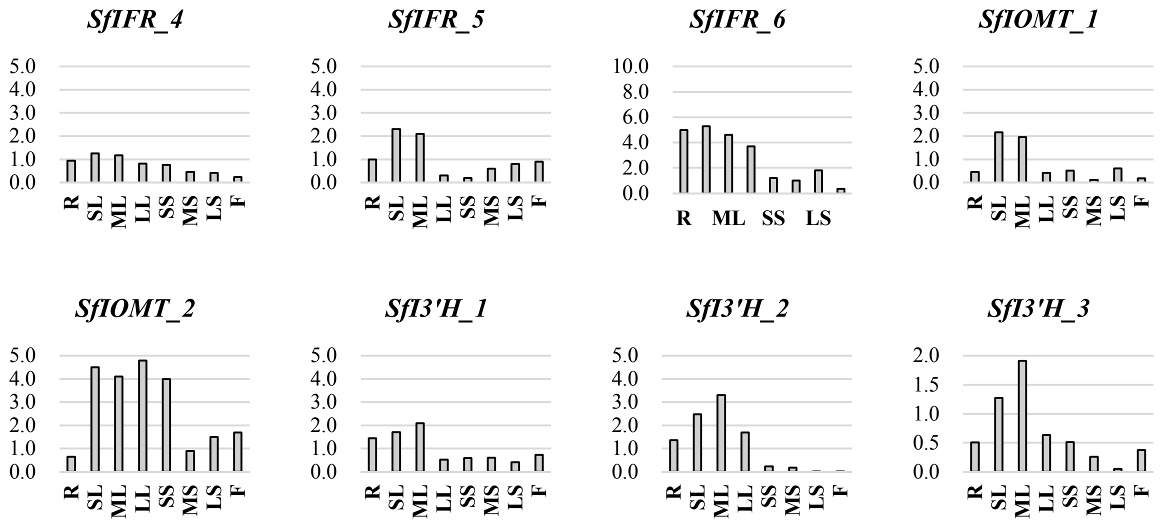

Supplementary Figure 1. Relative level of the flavonoid biosynthesis genes in root, leaves, stems and flower of Sophorae Radix. (a) Phenylpropanoid biosynthetic genes. (b) Phenolic acid biosynthetic genes. (c) Flavonoid biosynthetic genes. (d) Isoflavonoid biosynthetic genes. Transcript levels were determined after normalization with *Actin11* as the reference gene. Data are means of relative band intensities of three replicates and standard deviation (SD). R, root; SL, small leaf; ML, medium leaf; LL, large leaf; SS, small stem; MS, medium stem; LS, large stem; F, flower.

Supplementary Table 1 : Flavonoids contents in different organs of Sophorae Radix (μg/g dry weight). R, roots; SL, small leaves; ML, medium leaves; LL, large leaves; SS, small stems; MS, medium stems; LS, large stems; F, flowers.

Component		R	SL	ML	LL	SS	MS	LS	F
Phenylpropanoids	*t*-Cinnamic acid	ND	ND	ND	ND	0.62±0.01	0.58±0.03	0.46±0.01	ND
	p-Coumaric acid	ND	8.57±0.43	10.28±0.29	9.11±0.14	4.75±0.14	5.47±0.10	2.73±0.03	ND
Phenolic acid	Benzoic acid	ND	ND	ND	ND	121.83±2.16	43.52±3.94	64.25±2.58	29.12±0.48
	Caffeic acid	6.62±0.21	9.26±1.04	12.67±0.44	10.44±0.44	7.47±0.06	10.60±0.40	8.75±0.07	6.75±0.07
	Ferulic acid	8.79±0.14	6.61±0.05	5.95±0.26	6.32±0.17	7.95±0.13	5.72±0.17	5.40±0.04	7.16±0.14
	Chlorogenic acid	ND	ND	ND	ND	115.94±0.46	88.58±0.69	66.07±0.29	ND
Flavonoid	Kaempferol	36.91±7.82	ND	ND	ND	2.23±0.03	4.67±0.63	4.23±0.25	3.10±0.44
	Catechin hydrate	ND	ND	ND	ND	17.56±1.20	13.86±2.89	17.29±0.57	ND
	Epicatechin	ND	32.16±3.12	20.80±0.51	80.78±4.34	55.66±4.38	28.20±1.92	30.53±0.85	ND
	Rutin	261.03±10.07	352±1.02	5.46±0.15	6.3±0.08	ND	ND	ND	4.8±0.20
Isoflavonoid	Maackiain	218.17±22.04	ND	ND	23.59±5.14	ND	ND	ND	ND

*ND; not detected

Supplementary Table 2 : Blast search results of flavonoid biosynthetic genes in Sophorae Radix.

**Query**				**Subject**							
				**Sophorae Radix**		**tBLASTN results**					
**Gene**	**Origin species**	**GenBank accession no.**	**Transcript length (bp)**	**Transcript ID**	**Transcript length (bp)**	**Identity[a] (%)**	**Protein coverage (%)**	**Match length (Protein level)**	**Mismatch length (Protein level)**	**Gap**	**E-value**
PAL	*Glycine max*	NM_001250027	2,284	Sf102447_c0_g1_i1	2,644	91.0	98.7	713	58	2	0.00E+000
CNL	*Arabidopsis thaliana*	NM_105260	1,899	Sf115103_c0_g2_i1	2,219	60.5	96.7	579	200	6	0.00E+000
C4H	*Glycine max*	NM_001250388	1,531	Sf119713_c0_g1_i1	4,182	92.1	99.8	506	39	1	0.00E+000
				Sf110063_c0_g1_i1	1,945	65.8	92.9	474	155	4	0.00E+000
C3H	*Arabidopsis thaliana*	NM_180006	1,885	Sf108373_c0_g1_i1	2,131	82.9	96.5	491	84	0	0.00E+000
COMT	*Glycine max*	XM_003526719	1,353	Sf126433_c1_g1_i1	1,635	91.8	99.7	365	30	0	0.00E+000
	Glycine max	XM_003554738	1,390	Sf127741_c0_g1_i3	1,387	79.9	94.1	354	66	4	0.00E+000
				Sf127741_c0_g1_i2	1,533	76.3	99.7	379	81	6	0.00E+000
4CL	*Glycine max*	NM_001249489	1,869	Sf125371_c0_g2_i1	2,167	82.3	98.7	543	92	2	0.00E+000
	Glycine max	NM_001249307	1,697	Sf65872_c0_g1_i1	2,339	85.1	98.8	564	75	3	0.00E+000
CHS	*Glycine max*	NM_001317656	1,443	Sf115389_c2_g1_i2	1,672	93.0	99.5	388	27	0	0.00E+000
	Glycine max	XM_003533759	1,771	Sf131019_c3_g1_i1	1,412	74.8	99.7	389	97	1	0.00E+000
	Glycine max	XM_003531172	1,473	Sf101619_c0_g1_i1	1,847	85.0	99.2	387	57	1	0.00E+000
CHR	*Glycine max*	NM_001249578	1,167	Sf110292_c0_g1_i1	2,344	84.5	98.4	310	48	0	0.00E+000
				Sf120988_c1_g1_i1	1,816	71.0	99.4	314	90	1	6.00E-168
				Sf65645_c0_g2_i1	1,497	62.8	99.4	317	113	4	1.00E-138
CHI	*Glycine max*	NM_001249826	924	Sf94496_c0_g1_i1	873	84.5	99.6	226	33	1	2.00E-133
	Glycine max	NM_001249839	681	Sf113915_c0_g1_i1	1,373	77.3	95.1	216	49	0	2.00E-112
	Glycine max	NM_001248290	1,157	Sf127219_c1_g1_i1	1,383	82.3	98.2	215	38	0	3.00E-112
F3H	*Glycine max*	NM_001249868	1,405	Sf101281_c0_g2_i1	1,203	80.5	99.7	375	62	2	0.00E+000
F3'H	*Glycine max*	NM_001250086	1,585	Sf113707_c0_g1_i1	1,870	83.3	91.6	472	75	3	0.00E+000
FLS	*Glycine max*	NM_001250490	1,208	Sf129219_c0_g1_i2	1,167	85.1	99.7	335	49	1	0.00E+000
	Glycine max	XM_003521781	1,401	Sf99963_c0_g1_i1	1,321	72.0	98.0	343	90	4	0.00E+000
				Sf86503_c1_g1_i1	2,318	55.7	94.6	332	136	6	2.00E-124
				Sf112997_c2_g1_i1	2,405	55.7	94.6	332	136	6	5.00E-124

Supplementary Table 2 (continued)

Query				Subject							
				Sophorae Radix		tBLASTN results					
Gene	Origin species	GenBank accession no.	Transcript length (bp)	Transcript ID	Transcript length (bp)	Identity[a] (%)	Protein coverage (%)	Match length (Protein level)	Mismatch length (Protein level)	Gap	E-value
UFGT	*Arabidopsis thaliana*	AY114654	1,372	Sf126795_c1_g1_i2	2,008	50.6	99.1	484	210	12	1.00E-128
DFR	*Glycine max*	NM_001251683	1,279	Sf69410_c0_g1_i1	1,284	83.6	94.8	330	54	0	0.00E+000
LAR	*Glycine max*	XM_003556213	1,449	Sf124159_c0_g3_i6	1,412	50.8	82.7	304	180	2	1.00E-066
ANS	*Glycine max*	AY382829	1,252	Sf64126_c0_g1_i1	1,244	86.0	99.1	350	49	0	0.00E+000
ANR	*Cicer arietinum*	XM_004504890	1,300	Sf97139_c0_g1_i1	1,590	78.6	99.1	336	69	1	0.00E+000
IFS	*Glycine max*	NM_001249093	1,625	Sf107198_c1_g1_i1	2,169	82.4	96.4	505	83	2	0.00E+000
IFR	*Glycine max*	NM_001250708	1,113	Sf124159_c0_g3_i6	1,412	84.3	89.4	306	48	0	0.00E+000
				Sf85916_c1_g1_i1	1,385	79.8	98.1	307	60	1	5.00E-176
		NM_001251616	1,204	Sf90287_c0_g1_i2	1,471	82.5	99.7	308	53	1	0.00E+000
				Sf126666_c0_g1_i2	3,119	51.5	98.7	307	146	2	1.00E-094
				Sf119343_c0_g1_i3	1,874	73.0	97.5	311	84	0	2.00E-168
IOMT	*Glycine max*	XM_003542667	1,395	Sf97874_c1_g1_i1	2,274	83.9	96.4	353	52	2	0.00E+000
				Sf106043_c0_g1_i1	2,545	83.9	96.4	353	52	2	1.00E-179
I3'H	*Glycine max*	XM_003532623	1,652	Sf64464_c0_g1_i1	2,136	76.2	93.6	470	111	1	0.00E+000
				Sf124770_c0_g1_i1	1,997	69.6	91.8	461	139	1	0.00E+000
				Sf119131_c2_g1_i1	1,888	65.4	92.2	462	160	0	0.00E+000
ACT11	*Glycine max*	NM_001252731	1,372	Sf128341_c1_g1_i1	1,799	95.5	99.7	377	17	0	0.00E+000

[a]Amino acid sequence identity to the query protein

Supplementary Table 3 : Primers used in this study for semi-quantitative RT-PCR of flavonoid biosynthetic genes.

Gene	*Sophora flavescens* Transcript ID	Primer	Primer sequence (5' to 3')	Tm (°C)	Product size (bp)	Designation*
PAL	Sf102447_c0_g1_i1	Forward	ATCTCTCAGGTGGCTGCCAT	60.0	328	*SfPAL*
		Reverse	TGCCTGAGTACCCTTGGAGG	59.9		
CNL	Sf115103_c0_g2_i1	Forward	TTTTAATAATGGTTGGTTTCACACT	54.9	237	*SfCNL*
		Reverse	TTCCTACAATAGCCAATTATTTCA	53.3		
C4H	Sf119713_c0_g1_i1	Forward	GGTCACTCACCCACTCC	55.0	379	*SfC4H_1*
		Reverse	GTGAGGTTGCGGTGGTTG	58.0		
	Sf110063_c0_g1_i1	Forward	ACCATGCACTTAGCCTGATG	57.0	207	*SfC4H_2*
		Reverse	AGAGATGGGGAAAGTGGCA	57.2		
C3H	Sf108373_c0_g1_i1	Forward	CATTCCTTCACTTGGGGCCC	60.0	319	*SfC3H*
		Reverse	CCCACCAACCCCACTAATGC	60.0		
COMT	Sf126433_c1_g1_i1	Forward	ACAAGGGGTGTGGTTCACAT	58.4	264	*SfCOMT_1*
		Reverse	TATCACATTTTCCGGGAAGG	54.1		
	Sf127741_c0_g1_i3	Forward	CAATTAGGTTTTTGTCATCATGAAG	54.5	219	*SfCOMT_2*
		Reverse	CCGTGCTTTCCATGTCGCTG	61.3		
	Sf127741_c0_g1_i2	Forward	CAAGGTTTTGGTCATCATCATG	55.0	219	*SfCOMT_3*
		Reverse	CTGTGCTTTCCATGTCACTT	55.3		
4CL	Sf125371_c0_g2_i1	Forward	AACAGGGTTGCCAAAAGGGG	60.1	400	*Sf4CL2*
		Reverse	GGGGAATTTGGCCCTGACAG	60.0		
	Sf65872_c0_g1_i1	Forward	GCGTGCTTCCGTTGTTTCAC	60.0	369	*Sf4CL4*
		Reverse	GGAACGGCTGCTTTGCAAAG	60.0		
CHS	Sf115389_c2_g1_i2	Forward	TGAGCCTTGCGGATCTCAGA	60.0	221	*SfCHS*
		Reverse	AGCTACCTTCCCATCCACCC	60.0		
	Sf131019_c3_g1_i1	Forward	AACCCTCGGCATCGAAATGG	59.8	325	*SfCHS1*
		Reverse	TGGTACCTCCACAACCAGCA	60.1		
	Sf101619_c0_g1_i1	Forward	GCCGTGTGGTCCTAAGCTTC	60.1	272	*SfCHS3*
		Reverse	GCTTGTTTCGGCATCCCAGA	60.0		
CHR	Sf110292_c0_g1_i1	Forward	TACCACCATCCCTCCTGCAG	60.0	382	*SfCHR_1*
		Reverse	GAGTTGGGGCTTGGTTGGTC	60.2		
	Sf120988_c1_g1_i1	Forward	GCTTTAAGGGTGGTCCTGCC	60.0	333	*SfCHR_2*
		Reverse	TTGCTGCCATTTCCGCTCTT	59.9		
	Sf65645_c0_g2_i1	Forward	ATGGGCCTCTGCAATCTCCT	60.0	348	*SfCHR_3*
		Reverse	CCTGGAAACTGGGAACTGCC	59.9		
CHI	Sf94496_c0_g1_i1	Forward	TGACACCTTCCTTACGTATT	52.7	220	*SfCHI1B1*
		Reverse	CAGGGGTGAGAGGGTTGGAA	60.1		
	Sf113915_c0_g1_i1	Forward	TCCTCTGGGCAACACTGTGA	60.1	341	*SfCHI2A*
		Reverse	TCAAGTTCACCGCCATTGGG	59.9		
	Sf127219_c1_g1_i1	Forward	TCTGCAAATTGTTCAATGGC	54.5	330	*SfCHI*
		Reverse	CCCCTCCAGGATCCTCCAAG	60.1		
F3H	Sf101281_c0_g2_i1	Forward	GTGCCAGGATCAGTGTGTCG	60.1	345	*SfF3H*
		Reverse	GTGGCAAAAAGGGCGGTTTC	59.9		
F3'H	Sf113707_c0_g1_i1	Forward	CGACGTTGTAGTGGCAGCTT	60.0	359	*SfF3'H*
		Reverse	CGTCGTTGAACACTCGTCGT	60.0		
FLS	Sf129219_c0_g1_i2	Forward	GCACAAGAGCTTCAAAACAGG	57.3	226	*SfFLS_1*
		Reverse	ATGCACGAGTTTCCCTTCAT	56.5		
	Sf99963_c0_g1_i1	Forward	ATGGTGGGTTTGAAGGCCAC	59.8	359	*SfFLS_2*
		Reverse	TCGACCTCCATGGCCTTGAT	60.0		
	Sf86503_c1_g1_i1	Forward	TCAAAGACGGCAAGTGGCTC	59.9	205	*SfFLS_3*
		Reverse	GTGAGTGGTTTTGCAGGGCT	60.1		
	Sf112997_c2_g1_i1	Forward	AACCATCCACGGCCAACATC	60.0	261	*SfFLS_4*
		Reverse	GTGCCATCATTGGACAGGGG	60.1		
UFGT	Sf126795_c1_g1_i2	Forward	TGCCTCGGATTCCAACTCCT	59.9	154	*SfUFGT*
		Reverse	TTTGCTCTCGCCACATGACC	60.0		
DFR	Sf69410_c0_g1_i1	Forward	GTCCATGTTGTGCTGTTTGG	56.9	278	*SfDFR*
		Reverse	CCAAGGACCCTGAGAATGAA	55.8		
LAR	Sf124159_c0_g3_i6	Forward	CCACAGCATGAACCCTGTCC	60.0	286	*SfLAR*
		Reverse	CATCCCACCTTCCTCCTCGT	60.0		
ANS	Sf64126_c0_g1_i1	Forward	GAATCTGGGACACATTTTGC	54.3	202	*SfANS*
		Reverse	AGAAGGAAGTTGGGGGTATG	55.5		
ANR	Sf97139_c0_g1_i1	Forward	TCGACCTGAAGCAGATTCTC	56.1	315	*SfANR*
		Reverse	TTGTACGCTACAAAGCCACA	56.8		
IFS	Sf107198_c1_g1_i1	Forward	CAAATCAAGGGTCTTGTTGTG	54.7	238	*SfIFS*
		Reverse	CAACAGGTAGTGGTGGGTG	56.7		

Supplementary Table 3 (continued)

Gene	*Sophora flavescens* Transcript ID	Primer	Primer sequence (5' to 3')	Tm (°C)	Product size (bp)	Designation*
IFR	Sf124159_c0_g3_i6	Forward	CCACAGCATGAACCCTGTCC	60.0	284	*SfIFR_1*
		Reverse	CATCCCACCTTCCTCCTCGT	60.0		
	Sf85916_c1_g1_i1	Forward	CTGGGCTAATGTGGGGAGGA	60.0	235	*SfIFR_2*
		Reverse	GCAACTTGCCGATCAGGTCA	60.0		
	Sf90287_c0_g1_i2	Forward	GAGGTTCTGTGGCTTCTGGC	60.0	236	*SfIFR_3*
		Reverse	GAAGCTCGTTGAAGCGGTCA	60.0		
	Sf126666_c0_g1_i2	Forward	GGGGCAACAGGAAATCTGGG	60.0	188	*SfIFR_4*
		Reverse	AGCCTCAACAATGCTGGCTT	59.6		
	Sf119343_c0_g1_i3	Forward	GAGGAGGATTTGGTGGCTGC	60.1	348	*SfIFR_6*
		Reverse	AGGGCCAAGGACACACATCT	60.1		
IOMT	Sf97874_c1_g1_i1	Forward	GCCGCCTTTGTTTATTCTCT	55.5	326	*SfIOMT_1*
		Reverse	CCATGGAGCTAACGAAGTTG	55.6		
	Sf106043_c0_g1_i1	Forward	CCGCACAAAATAACAACCAT	54.2	265	*SfIOMT_2*
		Reverse	ATTTCACTTCAGAGCCGTTG	54.4		
I3'H	Sf64464_c0_g1_i1	Forward	CTGTGCCAGCTTCTGCATCA	60.0	284	*SfI3'H_1*
		Reverse	ATTCCCTCTGGTTCGGCTCA	59.9		
	Sf124770_c0_g1_i1	Forward	TGTGCTTCCCACTTCGATGC	60.0	296	*SfI3'H_2*
		Reverse	GGTGCAAGTTAGGTCGAGGC	60.1		
	Sf119131_c2_g1_i1	Forward	GATATGCTTGCCGGCGAGAA	59.9	227	*SfI3'H_3*
		Reverse	CACCAGGTCCACCCTCTCTT	59.8		
ACT11	Sf128341_c1_g1_i1	Forward	TTGGTCGACCTCGCCATACT	60.0	321	*SfACT11*
		Reverse	GGGAGAGGACAGCCTGGATT	60.0		

*Transcript variants are designated according to the name of the query genes and the designations are used in Figure 2. The order of the transcripts followed the sequence of flavonoid biosynthetic pathway.
